# eNOS Activation by HDL Is Impaired in Genetic CETP Deficiency

**DOI:** 10.1371/journal.pone.0095925

**Published:** 2014-05-15

**Authors:** Monica Gomaraschi, Alice Ossoli, Silvia Pozzi, Peter Nilsson, Angelo B. Cefalù, Maurizio Averna, Jan Albert Kuivenhoven, G. Kees Hovingh, Fabrizio Veglia, Guido Franceschini, Laura Calabresi

**Affiliations:** 1 Center E. Grossi Paoletti, Dipartimento di Scienze Farmacologiche e Biomolecolari, Università degli Studi di Milano, Milano, Italy; 2 Department of Clinical Sciences, University Hospital, Malmö, Sweden; 3 Department of Internal Medicine and Medical Specialties, Policlinico “Paolo Giaccone”, University of Palermo, Palermo, Italy; 4 University Medical Center Groningen, Groningen, The Netherlands; 5 Department of Vascular Medicine, Academic Medical Center, Amsterdam, The Netherlands; 6 Istituto Cardiologico Monzino, Milano, Italy; University of Bari & Consorzio Mario Negri Sud, Italy

## Abstract

Mutations in the *CETP* gene resulting in defective CETP activity have been shown to cause remarkable elevations of plasma HDL-C levels, with the accumulation in plasma of large, buoyant HDL particles enriched in apolipoprotein E. Genetic CETP deficiency thus represents a unique tool to evaluate how structural alterations of HDL impact on HDL atheroprotective functions. Aim of the present study was to assess the ability of HDL obtained from CETP-deficient subjects to protect endothelial cells from the development of endothelial dysfunction. HDL isolated from one homozygous and seven heterozygous carriers of CETP null mutations were evaluated for their ability to down-regulate cytokine-induced cell adhesion molecule expression and to promote NO production in cultured endothelial cells. When compared at the same protein concentration, HDL and HDL3 from carriers proved to be as effective as control HDL and HDL3 in down-regulating cytokine-induced VCAM-1, while carrier HDL2 were more effective than control HDL2 in inhibiting VCAM-1 expression. On the other hand, HDL and HDL fractions from carriers of CETP deficiency were significantly less effective than control HDL and HDL fractions in stimulating NO production, due to a reduced eNOS activating capacity, likely because of a reduced S1P content. In conclusion, the present findings support the notion that genetic CETP deficiency, by affecting HDL particle structure, impacts on HDL vasculoprotective functions. Understanding of these effects might be important for predicting the outcomes of pharmacological CETP inhibition.

## Introduction

Epidemiologic studies have clearly shown that high density lipoprotein cholesterol (HDL-C) levels are a strong, independent risk factor for the development of atherosclerotic coronary heart disease (CHD). Raising HDL-C has thus been proposed as a novel therapeutic strategy to reduce the significant burden of residual CHD in patients treated with lipid-lowering therapies [1]. Inhibitors of the cholesteryl ester transfer protein (CETP) have offered great promise as therapeutic means to raise plasma HDL-C levels [2]–[4]. However, large randomized trials with two CETP inhibitors failed to show a beneficial effect of the drugs in reducing cardiovascular events [5], [6]. These unexpected results have been ascribed to either off-target effects of the drug [5], [7],[8], to weak CETP inhibition and HDL-C raising activity [6], or to mechanism-related effects [9].

Besides their major role in promoting cell cholesterol efflux and reverse cholesterol transport [10], [11], HDL may exert atheroprotective activity by preventing endothelial dysfunction [12], a key step in the development of atherosclerosis. HDL downregulate cytokine-induced expression of cell adhesion molecules (CAMs) [12], and increase endothelial nitric oxide synthase (eNOS) expression and activation [13], NO release and bioavailability [14]. Impaired endothelial function has been reported in patients with genetic HDL deficiency [15], and the elevation of plasma HDL-C concentration in patients with low HDL-C levels by either niacin treatment or infusion of synthetic HDL leads to a significant improvement of endothelial function [15], [16].

Mutations in the *CETP* gene resulting in defective CETP activity have been shown to cause remarkable elevations of plasma HDL-C levels [17], with the accumulation in plasma of large, buoyant HDL particles enriched in apolipoprotein E (apoE) [18], similar to those produced by pharmacological CETP inhibition [19]. Genetic CETP deficiency thus represents a unique tool to understand the role of CETP on HDL function, and to evaluate the putative effects of CETP inhibition on HDL function without potential off-target effects of CETP inhibitors. Indeed, both genetic and pharmacological CETP inhibition enhances HDL capacity to promote cholesterol efflux from macrophages, likely through the formation of apoE-rich particles [18]–[20]. Little is known on the effect of pharmacological or genetic CETP inhibition on HDL capacity to prevent endothelial dysfunction [21]. The present study was undertaken to evaluate the ability of HDL obtained from CETP-deficient subjects to protect endothelial cells from the development of endothelial dysfunction.

## Materials and Methods

### Subjects

One homozygous and 7 heterozygous carriers of null CETP mutations belonging to three caucasian kindreds [22]–[24] volunteered for the study. The homozygote carries the R37X CETP mutation [22]; the 7 heterozygotes carry 3 different CETP mutations: R37X [22], Q165X [23], and IVS7+1 [24]. Age and sex matched healthy individuals were selected as controls among blood donors attending the Servizio Immunoematologico Trasfusionale of the Niguarda Hospital. The study was conducted according to the guidelines set out in the Declaration of Helsinki and was approved by the Ethic Committee of the Niguarda Hospital (approved on 12/09/2008), and all subjects signed an informed consent. Blood samples were collected after an overnight fast and plasma was prepared by low speed centrifugation at 4°C. Aliquots were immediately frozen and stored at −80°C until assayed.

Plasma total and HDL cholesterol, and triglycerides were measured by certified enzymatic techniques. LDL-C was calculated using the Friedewald’s formula. ApoA-I, apoA-II, and apoB levels were determined by immunoturbidimetry; the plasma concentration of HDL particles containing only apoA-I (LpA-I) and of particles containing both apoA-I and apoA-II (LpA-I:A-II) was determined by electroimmunodiffusion in agarose gel (Sebia Italia). Plasma CETP concentrations were measured by competitive ELISA [22]. CETP activity was measured with a fluorometric assay kit (ROAR Biomedical Inc, New York, NY, USA). Plasma levels of the soluble forms of vascular cell adhesion molecule 1 (VCAM-1), intracellular cell adhesion molecule 1 (ICAM-1) and E-selectin were determined by commercial ELISA kits (R&D Systems, Minneapolis, MN, USA).

### Lipoprotein Preparation and Characterization

Total HDL (d = 1.063–1.21 g/ml), HDL2 (d = 1.063–1.125 g/ml) and HDL3 (d = 1.125–1.21 g/ml) were isolated by sequential ultracentrifugation. Total HDL, HDL2, and HDL3 were separated according to size by non-denaturing polyacrylamide gradient gel electrophoresis (GGE) [22], and according to size and charge by 2D electrophoresis and subsequent immunodetection with anti apoA-I or anti apoE antibodies [22]. ApoE-containing particles were precipitated from the HDL2 ultracentrifugal fraction by the heparin-MnCl_2_ method [25]. Lipoproteins were dialyzed against sterilized saline immediately before use and their concentrations are expressed as mg of protein/ml. The concentration of sphingosine-1-phoshate (S1P) in isolated HDL fractions was measured with a commercial competitive ELISA kit (Echelon Biosciences Inc., Salt Lake City, UT, USA) and normalized by protein concentration.

### HDL Activity in Cultured Endothelial Cells

Primary cultures of human umbilical vein endothelial cells (HUVEC) were purchased from Clonetics (Lonza, Milano, Italy) and subcultured for 1–3 passages according to manufacturer instructions. Experiments were performed in M199 with 0.75% BSA and 1% FCS. HDL, HDL2 and HDL3 fractions were used at the protein concentration of 1.0 mg/ml in all experiments.

To investigate the ability of HDL to downregulate cytokine-induced VCAM-1 expression, cells were incubated overnight with HDL, HDL2, or HDL3, washed with PBS to remove lipoproteins, and stimulated with tumor necrosis factor alpha (TNFα) (10 ng/ml) for 8 hours. VCAM-1 concentration in conditioned media, which reflects VCAM-1 cell expression [26], was evaluated using the CytoSets™ ELISA kit (BioSource International, Camarillo, CA, USA), and normalized by the protein concentration of total cell lysate.

To investigate the effects of HDL on NO production, cells were incubated with HDL, HDL2, or HDL3 for 30 minutes and NO levels were measured by fluorescence using a diacetate derivative of 4,5-diaminofluorescein (DAF-2 DA, Sigma-Aldrich Chemie, Steinheim, Germany). For each sample, fluorescence was normalized by the protein concentration of total cell lysate. To investigate HDL effects on eNOS activation by phosphorylation, cells were incubated with HDL, HDL2 or HDL3 for 10 minutes. Proteins were separated by SDS-PAGE and then transferred on a nitrocellulose membrane. Membranes were developed against phosphorylated eNOS (Ser1177, Cell Signalling Technology, Beverly, MA, USA), stripped and reprobed with an antibody against total eNOS. To investigate HDL effects on eNOS expression, cells were incubated overnight with HDL, HDL2 or HDL3. Proteins were separated by SDS-PAGE and transferred on a nitrocellulose membrane. Membranes were developed against total eNOS (BD Biosciences, San Jose, CA, USA), stripped and reprobed with an antibody against β-actin (Sigma-Aldrich Chemie). Bands on membranes were visualized by enhanced chemiluminescence (GE Healthcare Biosciences, Uppsala, Sweden). Band densities were evaluated with a GS-690 Imaging Densitometer and a Multi-Analyst software (Bio-Rad Laboratories, Hercules, CA, USA).

### Statistical Analyses

Results are reported as means±SD, if not otherwise stated. The association of plasma lipids, and CETP activity and mass, with CETP genotype was assessed by two different General Linear Models (GLM): (i) as the linear trend versus the number of mutant *CETP* alleles (0, 1, or 2) (model 1), or (ii) as the comparison between carriers and controls (model 2). The association of HDL functions with CETP genotype was assessed only by comparison between carriers and controls. Since we have only one homozygote, with an extreme phenotype in terms of HDL structure, and thus to be considered an outliner, the subject has been excluded from the analyses. Nevertheless, we have repeated the analyses including the homozygote to assess the stability of the results. All tests were two-sided and p-values <0.05 were considered as significant. All analyses were performed by using the SAS Statistical package v. 9.2 (SAS Institute Inc., Cary NC, USA).

## Results

### Plasma Lipids and Lipoproteins

Plasma lipid levels in the examined subjects are reported in Table 1. Plasma total and HDL cholesterol, apoA-I, LpA-I, and LpA-I:A-II levels were significantly higher, in a gene-dose dependent manner, in carriers of CETP mutations than controls. Plasma apoA-II levels also tended to increase with the number of mutant *CETP* alleles, but the differences did not reach statistical significance. Plasma LDL-C, triglyceride, and apoB levels were similar in carriers and controls. CETP activity and mass were null in the homozygous carrier and significantly reduced in heterozygotes.

**Table 1:** Plasma lipids and inflammatory markers.

	Carrier of2 mutantCETP alleles	Carriers of1 mutantCETP allele	All carriers	Controls	*P* (trend)	*P* (carriersvs.controls)
n.	1	7	8	8		
Age (y)	65	43.1±14.9	45.9±68.9	46.0±14.7		
Gender (M/F)	M	4M/3F	5M/3F	5M/3F		
Total cholesterol (mg/dl)	355	189.4±39.3	210.1±68.9	149.2±22.3	<0.001	0.03
LDL- cholesterol (mg/dl)	131	110.3±26.8	112.9±25.9	91.7±22.9	0.07	0.10
HDL-cholesterol mg/dl)	208	68.4±15.9	85.9±51.5	50.7±8.2	0.001	0.08
Triglycerides (mg/dl	79	82.4±49.0	82.0±45.4	61.0±23.3	0.32	0.26
Apolipoprotein A-I (mg/dl)	272	138.3±25.4	155.0±52.8	112.3±11.9	<0.001	0.043
Apolipoprotein A-II (mg/dl)	50	37.1±13.2	38.8±13.1	30.1±1.5	0.15	0.30
Apolipoprotein B (mg/dl)	77	88.7±18.3	87.3±17.5	86.7±14.6	0.80	0.94
LpA-I (mg/dl)	91	59.9±12.1	63.8±15.7	52.0±8.5	0.011	0.008
LpA-I:A-II (mg/dl)	181	78.4±18.7	91.3±40.6	60.3±16.2	<0.001	0.006
CETP activity (pmol/ml/h)	0	47.4±20.7	31.2±19.1	133.1±9.6	<0.001	<0.001
CETP mass (µg/ml)	0	1.1±0.2	1.0±0.4	1.4±0.2	<0.001	0.01
sVCAM-1 (ng/ml)	422	388.6±136.4	390.3±121.2	578.5±91.8	0.02	0.005
sICAM-1 (ng/ml)	209	195.2±30.7	196.9±28.8	264.8±49.3	0.02	0.005
sE-Selectin (ng/ml)	41	33.4±19.6	34.6±18.2	59.2±9.9	0.02	0.006

Data are expressed as mean±SD.

The plasma levels of the soluble forms of VCAM-1, ICAM-1, and E-Selectin were significantly lower in carriers of CETP mutations than in controls (Table 1). A negative correlation was observed in the entire cohort of examined subjects between plasma levels of HDL-C and soluble CAMs (R = −0.446, *P* = 0.096 for sVCAM-1; R = −0.478, *P* = 0.084 for sICAM-1; R = −0.752, *P* = 0.002 for sE-selectin).

**Figure 1:**
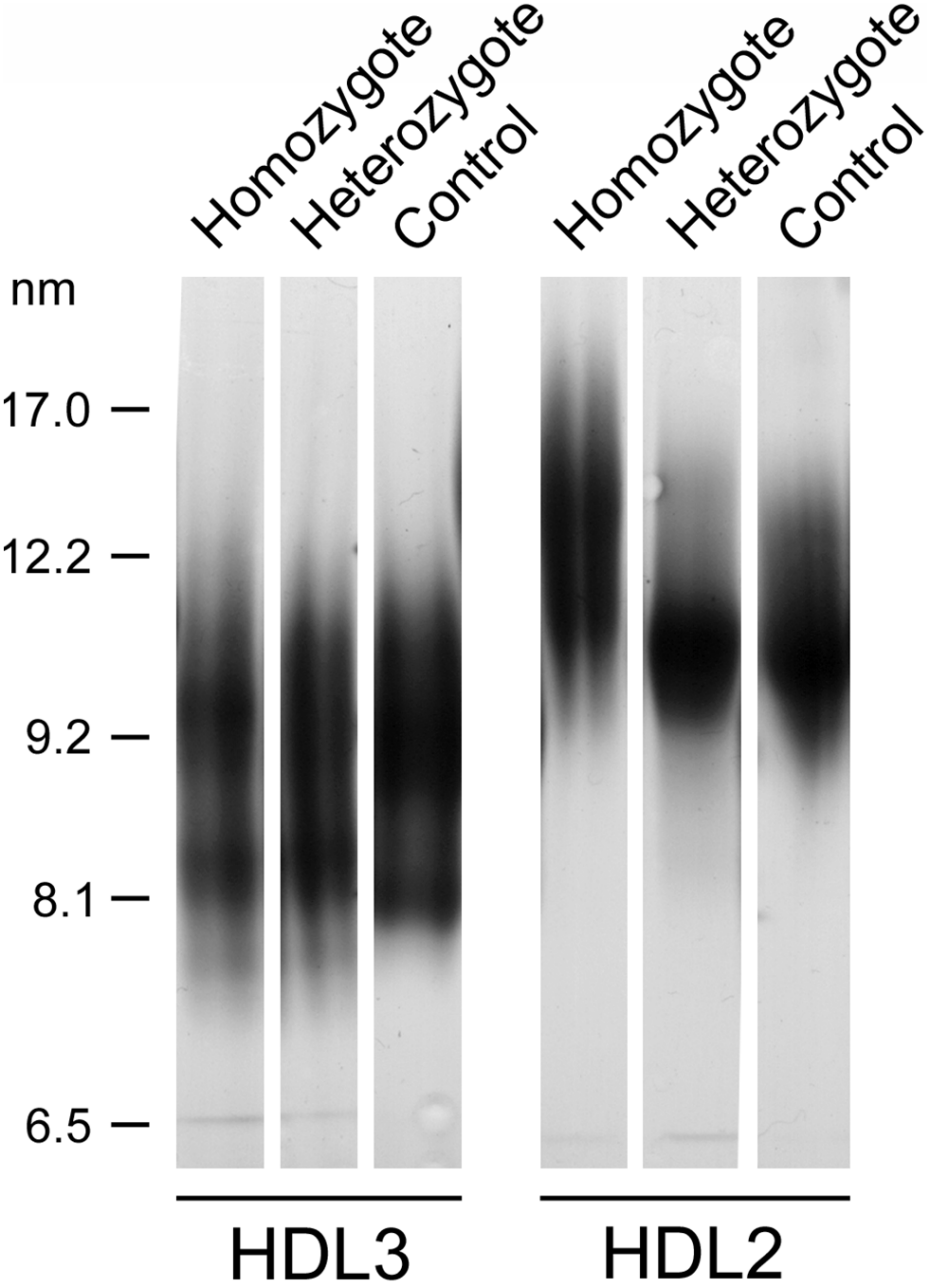
GGE analysis of purified HDL2 and HDL3. HDL fractions isolated from the homozygote, and a representative heterozygote and control were analyzed by GGE.

**Figure 2:**
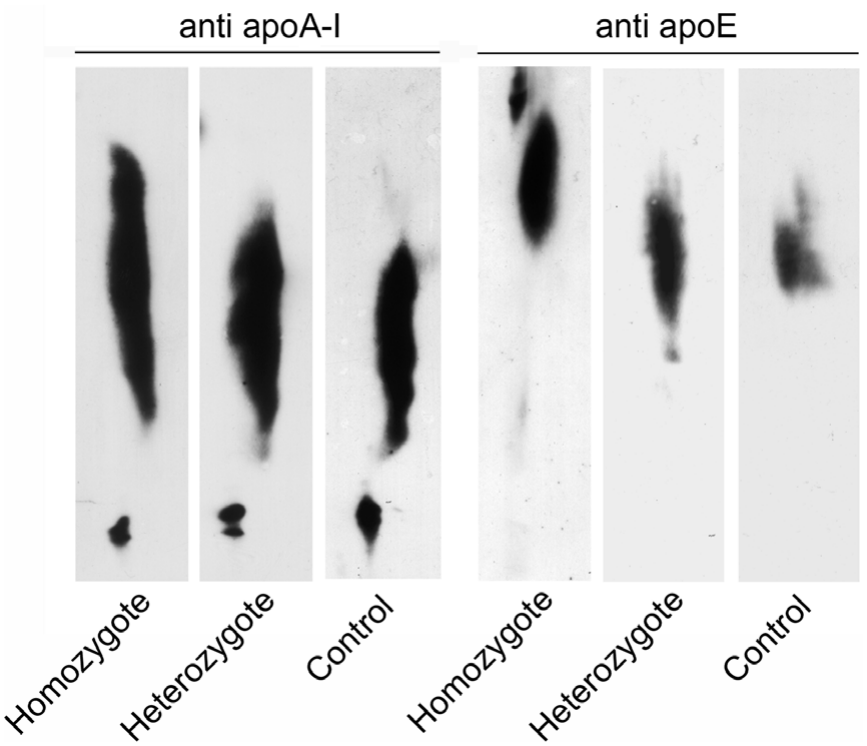
2D electrophoresis analysis of purified HDL. HDL isolated from the homozygote, and a representative heterozygote and control were separated by 2D electrophoresis and immunodetected with anti apoA-I and anti apoE antibodies.

The HDL2 fraction isolated from the homozygote consisted of two populations of particles with a diameter of 12.4 and 13.6 nm, i.e. distinct from control HDL2, which consisted of a single population of particles of 11.5 nm (Figure 1). The size of HDL2 isolated from heterozygotes (11.6 nm) was comparable to that of control HDL2. HDL3 particle size was very similar in all examined subjects (Figure 1). When examined by 2D electrophoresis, the larger HDL2 found in homozygous plasma appeared to be remarkably enriched in apoE (Figure 2).

### Effects of HDL on Cytokine-induced Endothelial VCAM-1 Expression

**Figure 3:**
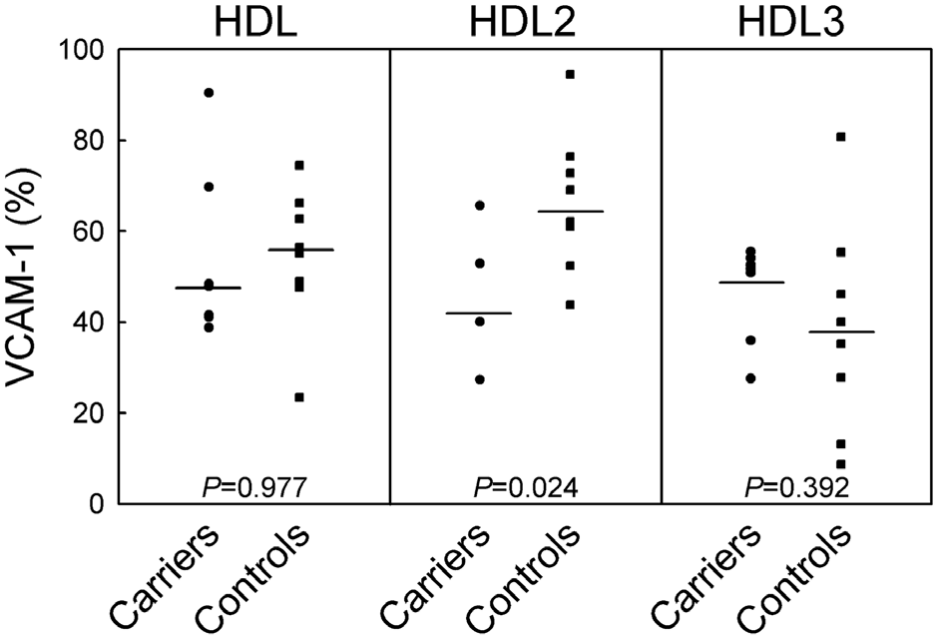
Effects of HDL isolated from carriers of CETP mutations and controls on VCAM-1 expression in TNFα-stimulated HUVEC. Cells were incubated overnight with HDL, HDL2, or HDL3 isolated from 7 heterozygous carriers of CETP mutations and age-sex matched controls (n = 8), at the concentration of 1.0 mg of protein/ml, before stimulation with TNFα for 8 hours. Results are expressed as percentage of VCAM-1 concentration in conditioned media of untreated TNFα-stimulated cells. Data points for each study participant are shown.

**Figure 4:**
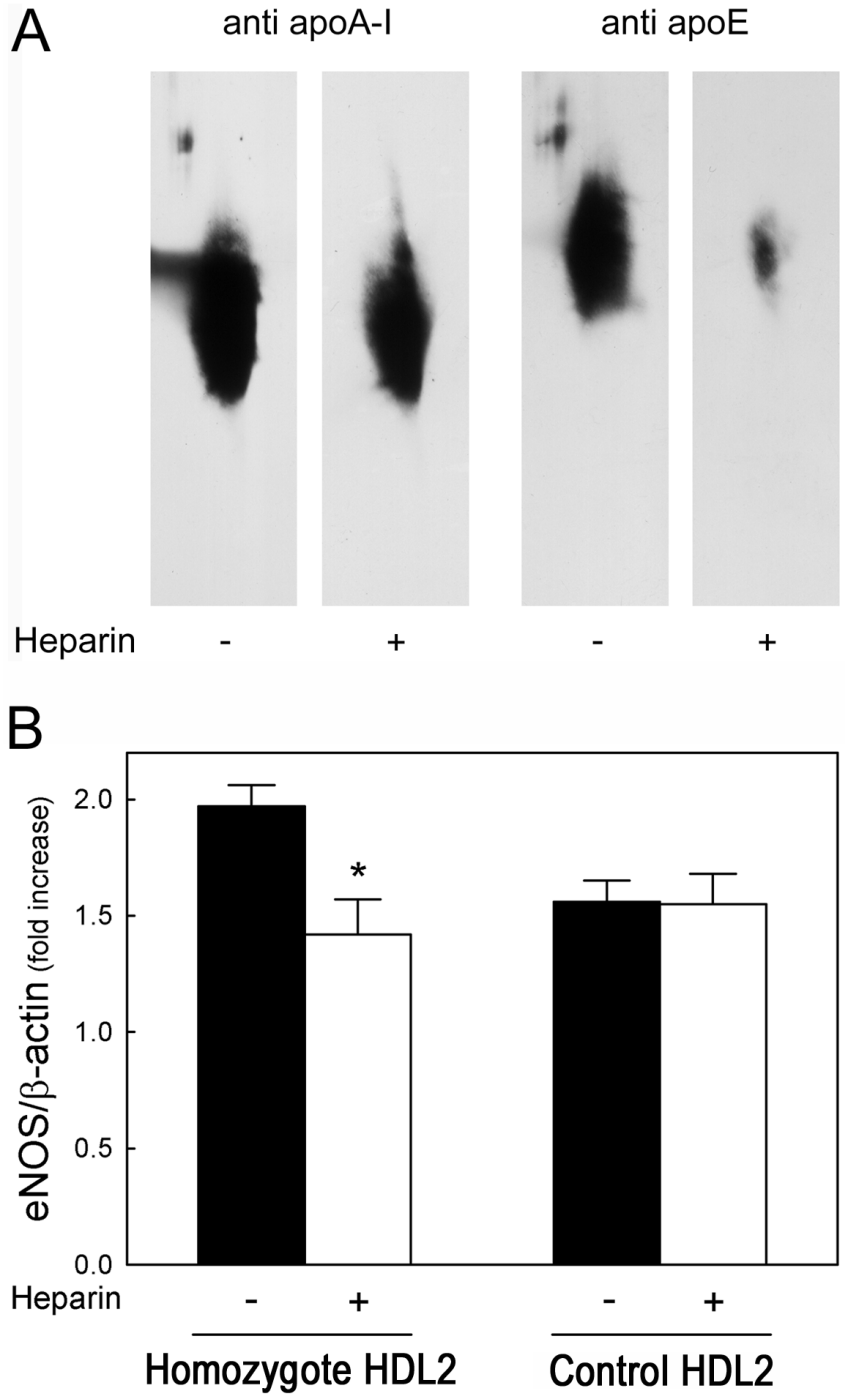
Effect of apoE depletion on eNOS expression in HUVEC. Panel A. HDL2 isolated from the homozygous carrier of the R37X CETP mutation were separated by 2D electrophoresis, followed by anti apoA-I or anti apoE immunodetection, before (−) and after (+) incubation with heparin-MnCl_2_. Panel B. Cells were incubated overnight with HDL2 (1 mg/ml) from the homozygous carrier of the R37X CETP mutation and from controls (n = 3) before (full bars) and after (open bars) treatment with heparin-MnCl_2_. Western blot analysis of eNOS protein was performed, and eNOS protein band intensities were normalized for β-actin values and expressed as fold of increase in treated vs. untreated.

HDL from controls remarkably down-regulated TNFα-induced VCAM-1 expression in HUVEC, with a significantly greater activity of HDL3 than HDL2 (Figure 3), as previously reported [27]. HDL and HDL3 from heterozygous carriers of CETP mutations were as effective as control HDL and HDL3 in inhibiting VCAM-1 expression, but HDL2 from carriers displayed a greater inhibitory activity than control HDL2 (Figure 3). No differences in the results were observed when the homozygote was included in the analysis. The greater anti-inflammatory activity of carrier than control HDL2 is unlikely due to the presence of large apoE-containing particles, as removal of these particles by precipitation with heparin-MnCl_2_ (Figure 4A) did not affect their capacity to downregulate cytokine-induced VCAM-1 expression (−61.7±12.2% and −63.9±1.4% with native and heparin-treated HDL2, respectively).

### Effects of HDL on NO Production and eNOS Activation

**Figure 5:**
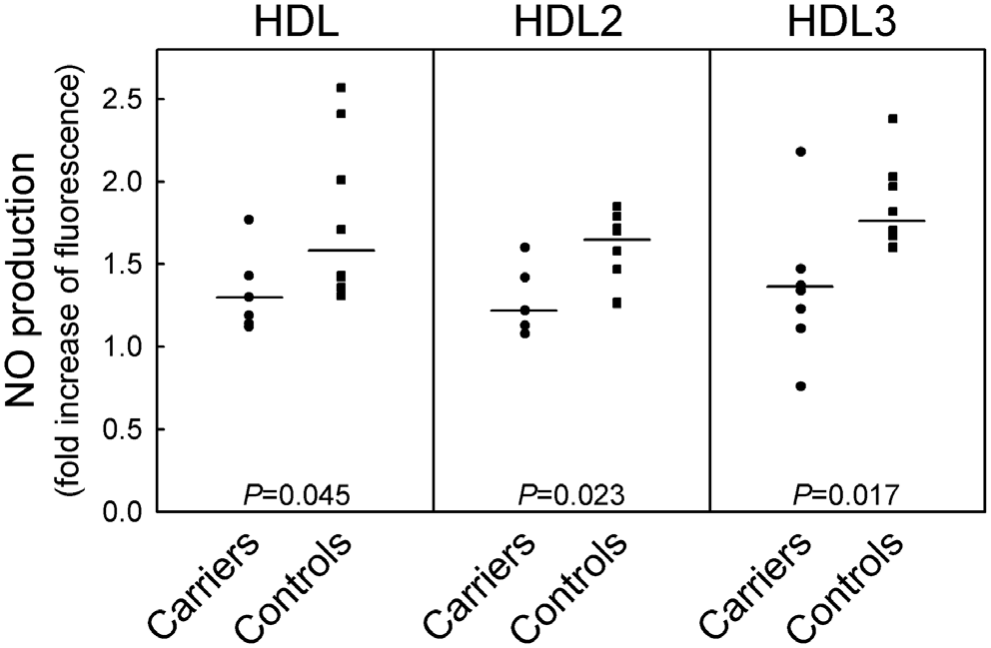
Effects of HDL isolated from carriers of CETP mutations and controls on NO production in HUVEC. Cells were incubated overnight with HDL, HDL2, or HDL3 isolated from 7 heterozygous carriers of CETP mutations and age-sex matched controls (n = 8), at the concentration of 1.0 mg of protein/ml. Results are expressed as fold of increased fluorescence in treated vs. untreated cells. Data points for each study participant are shown.

HDL obtained from control subjects stimulate NO production in HUVEC, and no significant difference between HDL2 and HDL3 fractions was observed (Figure 5). All HDL fractions isolated from heterozygous carriers of CETP mutations were less efficient than control HDL in inducing NO production (Figure 5). No differences in the results were observed when the homozygote was included in the analysis.

**Figure 6:**
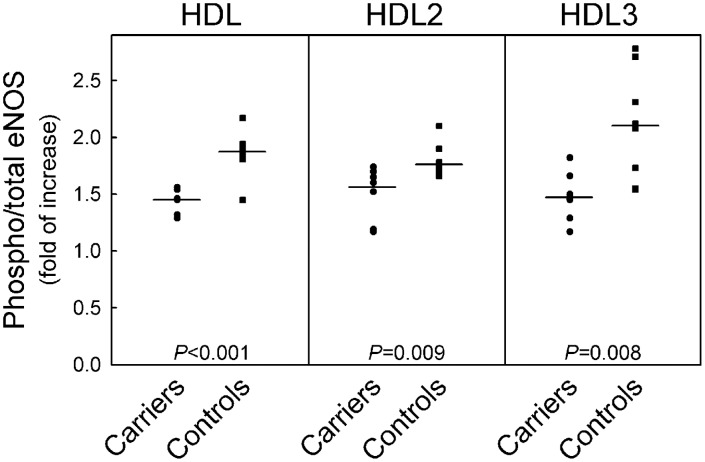
Effects of HDL isolated from carriers of CETP mutations and controls on eNOS activation in HUVEC. Cells were incubated for 10-sex matches controls (n = 8), at the concentration of 1.0 mg of protein/ml. Western blot analysis of the phosphorylated and total forms of eNOS was performed, and the phosphorylated/total eNOS ratios were calculated by densitometric analysis and expressed as fold of increase in treated vs. untreated cells. Data points for each study participant are shown. cells. Results are mean±SEM of 3 separate experiments performed with 1 preparation of homozygote HDL2, 3 preparations of control HDL2, and 3 batches of cells. *P<0.05 vs. untreated homozygote HDL2.

HDL from controls induced a marked activation of eNOS in HUVEC (Figure 6), with no difference between HDL2 and HDL3 fractions. All HDL fractions isolated from heterozygous carriers of CETP mutations showed a significantly reduced ability to activate eNOS than control HDL (Figure 6). No differences in the results were observed when the homozygote was included in the analysis. Since S1P within HDL was shown to increase their ability to activate eNOS [28], S1P levels were measured in HDL fractions from carriers of CETP mutations and controls. The concentrations of S1P in HDL and HDL3 from carriers were significantly lower compared to S1P concentration in control HDL and HDL3 (Table 2); S1P content of HDL2 was also lower in carriers than in controls, but this difference did not achieve statistical significance (Table 2).

**Table 2:** Sphingosine-1-phosphate levels in HDL, HDL2, and HDL3.

	Heterozygous Carriers	Controls	*P*
	S1P (pmol/mg of protein)		
HDL	188.2±67.3	290.6±92.3	0.05
HDL2	169.1±13.3	226.2±81.0	0.16
HDL3	158.3±97.8	312.0±54.7	0.012

Data are expressed as mean±SD.

To prove whether the reduced ability of HDL from carriers to induce NO production was due to the reduced S1P content, HDL from the homozygous carrier of the R37X mutation were also tested after the addition of 100 pmoles of S1P, an amount necessary to reach the S1P content of HDL from controls (295 pmol/mg protein). Indeed, the addition of S1P improved the ability of homozygote HDL to induce NO production from 1.25±0.07 to 1.61±0.08 fold increase (*P* = 0.028), a value comparable to that of HDL from controls (1.60±0.12 fold).

HDL from controls remarkably increased eNOS expression in HUVEC, as demonstrated by the 1.62±0.13 fold increase in eNOS protein. No difference was observed between HDL2 and HDL3 fractions (1.54±0.18 fold and 1.56±0.14 fold, respectively). HDL and HDL3 from heterozygous carriers of CETP mutations were as effective as control HDL and HDL3 in enhancing eNOS production (1.82±0.27 fold, *P* = 0.09 vs. control HDL, and 1.60±0.20 fold, *P* = 0.65 vs. control HDL3); by contrast, HDL2 from carriers caused a significantly greater increase in eNOS production than control HDL2 (1.99±0.08 fold, *P*<0.001 vs. control HDL2). No differences in the results were observed when the homozygote was included in the analysis. The enhanced capacity of carrier HDL2 to stimulate eNOS production appears to be related to the enrichment in apoE-containing particles, as their removal by precipitation with heparin-MnCl_2_ (Figure 4A) reduced eNOS induction from 1.97±0.09 to 1.42±0.15 fold (*P* = 0.035), i.e. very close to that of control HDL2, which was not affected by heparin-MnCl_2_ treatment (Figure 4B).

## Discussion

This study was undertaken to assess the ability of HDL isolated from subjects with genetic CETP deficiency to maintain endothelial cell homeostasis. The results demonstrate that HDL from carriers of CETP mutations are equally effective as control HDL in inhibiting cytokine-induced expression of VCAM-1 in cultured endothelial cells. Consistent with this *in vitro* finding, a proportionate reduction in the plasma concentration of soluble CAMs was found in association with the enhanced plasma HDL levels in CETP-deficient subjects. The effects of genetic CETP deficiency on the ability of HDL to induce NO bioavailability in cultured endothelial cells are complex since the enhanced capacity of HDL from CETP-deficient subjects to stimulate eNOS expression is offset by a reduced capacity to activate eNOS, resulting in a decreased NO production.

HDL ability to downregulate cytokine-induced CAM expression in endothelial cells has been widely recognized as part of their anti-inflammatory activity [12]. Here we show that HDL isolated from CETP-deficient subjects are as efficient as control HDL in inhibiting VCAM-1 expression. In control subjects, HDL3 are more effective than HDL2 in inhibiting endothelial VCAM-1 expression [27]. In CETP-deficient subjects, the slightly reduced capacity of HDL3 to inhibit VCAM-1 expression compared with control HDL3 is offset by a remarkably greater anti-inflammatory activity of HDL2. This latter effect is likely due to the peculiar protein and lipid composition of HDL from CETP-deficient subjects, which are enriched in apoA-I, and thus have a superior inhibitor capacity than particles enriched in apoA-II [29], and are depleted in triglycerides [30], which reduce HDL ability to down-regulate VCAM-1 expression [31].

HDL ability to stimulate NO production represents another vasculoprotective property of HDL [13]. Here we show that HDL from CETP-deficient subjects are less effective than control HDL in inducing NO production due to a reduced capacity to activate eNOS, despite an increased ability to stimulate eNOS expression. eNOS activation by HDL requires the interaction with both the scavenger receptor class B type I and the lysosphingolipid receptor S1P3 [13]. The protein component of HDL, mainly apoA-I, is necessary for the interaction with SR-BI, while minor bioactive components of HDL, such as lysophospholipids, and particularly S1P, are necessary to activate the S1P3 receptor [28]. All HDL fractions isolated from plasma of CETP-deficient subjects contain less S1P than HDL obtained from control subjects, which likely explains the reduced capacity to activate eNOS, as suggested by the finding that the addition of S1P to carrier HDL restores the impaired functionality. Moreover, HDL from CETP-deficient subjects are enriched in LpA-I:A-II particles, which have been shown to be less efficient than LpA-I in interacting with SR-BI [32], [33], and have a reduced content of PON1 [34], an enzyme recently suggested to play a role in eNOS activation [14], which may further contribute to the reduced functionality of carrier HDL. HDL from CETP-deficient subjects are instead more efficient than control HDL in enhancing the expression of eNOS. The enhanced eNOS expression has little effect on NO production *in vitro*, but may improve NO bioavailability *in vivo*, where eNOS could eventually be activated by a variety of stimuli [35]. HDL structural requirements for HDL-induced eNOS expression are largely unknown. Here we show that HDL2 from CETP-deficient subjects are very effective in enhancing eNOS production through an apoE-dependent pathway. The same HDL2 particles have also been shown to be more effective than control HDL2 in promoting cell cholesterol efflux via ABCG1, in a process also dependent on apoE [18], [20]. ABCG1 has been recently described as an important player in preserving endothelial homeostasis, as ABCG1 deficiency causes endothelial activation, which in turn promotes monocyte-endothelium interaction [36]. Moreover, ABCG1 is necessary for HDL-mediated vasculoprotection in mice fed a high-cholesterol diet [37]. One can speculate that the accumulation of apoE-rich HDL in genetic CETP deficiency is responsible for the increased capacity of these particles to enhance eNOS protein levels, a process likely mediated by apoE-facilitated cholesterol and oxysterols removal through ABCG1 [37].

The present findings may be relevant in the context of the current debate on the potential negative effects of pharmacological CETP inhibition on HDL function [9]. Previous studies have shown that both genetic and pharmacological CETP inhibition enhances HDL capacity to promote cholesterol efflux from macrophages [18]–[20], [38]. Here we show that genetic CETP deficiency does not affect the capacity of HDL to downregulate cytokine-induced CAMs expression, i.e. similar to what observed with HDL isolated from subjects treated with a potent CETP inhibitor [21]. Direct *in vivo* measurements of NO-dependent endothelial function in CETP-deficient subjects are warranted to understand the significance of the present *ex-vivo* findings on HDL capacity to promote NO production. Notably, pharmacological CETP inhibition has little effect on *in vivo* measures of endothelial function in humans [39], [40], which is consistent with the present *in vitro* findings.
